# High Prevalence and Overexpression of Fosfomycin-Resistant Gene *fos*X in *Enterococcus faecium* From China

**DOI:** 10.3389/fmicb.2022.900185

**Published:** 2022-07-08

**Authors:** Ling Xin, Xiaogang Xu, Qingyu Shi, Renru Han, Jue Wang, Yan Guo, Fupin Hu

**Affiliations:** ^1^Institute of Antibiotics, Huashan Hospital, Fudan University, Shanghai, China; ^2^Key Laboratory of Clinical Pharmacology of Antibiotics, Ministry of Health, Shanghai, China

**Keywords:** *Enterococci*, fosfomycin, resistance, *fos*X, prevalence

## Abstract

*Enterococc*i are one of the main causes of gastrointestinal tract infections in the healthcare system and can develop resistance to fosfomycin through plasmid or chromosomally encoded fosfomycin resistance genes. To investigate the mechanisms of fosfomycin resistance, a total of 4,414 clinical isolates of non-replicated clinical enterococci collected from 62 hospitals in 26 provinces or cities in China were tested. Antibiotic susceptibility testing, detection of fosfomycin resistance genes, and cloning of the *fos*X gene were done. The PFGE, MLST, qRT-PCR, and next genome sequencing were carried out. The results revealed that the fosfomycin-resistant rate of enterococci was 3.5% (153/4,414), and the major resistance mechanism was *fos*X (101/153) and *fos*B (52/153) genes. The *fos*X gene could increase 4- fold fosfomycin MIC in *Enterococcus faecium* BM4105RF transformants, and the results of PFGE showed the 101 *E. faecium* carrying *fos*X were grouped into 48 pulse types. The multilocus sequence typing identified ST555 as the vast majority of STs, mostly distributed in Shanghai, China. Furthermore, the *fos*X gene expression was strongly related to the fosfomycin-resistant levels of *enterococci*. The present study was the first to describe the high prevalence presence of the *fos*X gene in *E. faecium* from China.

## Introduction

*Enterococci* are significant opportunistic nosocomial pathogens of the gastrointestinal tract, occupying second place in the detection rate of Gram-positive bacteria (Fillgrove et al., 2003). Currently, clinically limited antibiotics are available for the treatment of enterococcal infections (Scortti et al., 2018). As a phosphoric acid antibiotic agent, fosfomycin showed broad-spectrum antibacterial activity and acted as the first step in the synthesis of bacterial peptidoglycan (Fillgrove et al., 2003; Chen et al., 2019; Zhang et al., 2020). With the widespread use of fosfomycin, the emergence of *enterococci* has become a major concern (Fillgrove et al., 2003; Scortti et al., 2018).

Fosfomycin resistance mechanisms have been proposed, including inherent the acquisition of chromosomal mutations and plasmid-encoded fosfomycin-modifying enzymes. Regarding the mechanisms of chromosomal mutations, it supported the replacement position of cysteine in the active site of the UDP-N-acetylglucosamine-3-enolpruvyltransferase (MurA), such as *Borrelia burgdorferi*, *Mycobacterium tuberculosis*, etc. (Fillgrove et al., 2003; Clinical and Laboratory Standards Institute, 2020). Moreover, fosfomycin resistance is also mediated by reduced antimicrobial uptake by chromosomal mutations in the *glp*T and *uhp*T genes (Clinical and Laboratory Standards Institute, 2020). Different fosfomycin-modifying enzymes had been described, including metalloenzymes (FosA, FosB, and FosX) (Murray et al., 1990; Bernat et al., 1997; Xu et al., 2013; Chen et al., 2014) and fosfomycin kinases (FomA, FomB, and FosC) (Mendoza et al., 1980; Bernat et al., 1997; Sahni et al., 2013).

Fillgrove et al. (2003) found a related subfamily enzyme named FosX with 30–35% similarity to the sequences of FosA and FosB proteins in the microbial genome sequence database. Scortti et al. (2018) reported that the FosX-mediated resistance is epistatically suppressed by two members of the PrfA virulence regulon, *hpt* and *prf*A, which upon activation by host signals induce increased fosfomycin influx into the bacteria cell. Chen et al. (2019) found that the aminoglycosides resistance gene *rmt*B may be co-disseminated with *bla*_*KPC–2*_ and *fos*A3 genes through plasmid, which results in fosfomycin-resistance isolates difficult-to-treat pathogen due to limited treatment options. Zhang et al. (2020) reported that the coexistence of *van* series genes and *fos* genes simultaneously can greatly improve the transfer efficiency in *Enterococcus faecium*.

At present, most of the previous studies investigated the mechanism of fosfomycin resistance among Gram-negative bacteria, and only limited information about the resistance mechanism of Gram-positive, particularly *enterococci*, is available. In the published articles, *fos*A, *fos*C, and *fos*X genes were detected in gram-negative bacteria, while *fos*B genes were detected in gram-positive bacteria. In this study, we collected 4,414 clinical isolates of enterococci collected from 62 hospitals in 26 provinces or cities across China from 2017 to 2020 and aimed to survey the prevalence of fosfomycin resistance and the associated *fos*B, *fos*X, and *mu*rA genetics in clinical isolates of enterococci in China.

## Materials and Methods

### Bacterial Strains

The bacteria in this research were collected from 62 hospitals in 26 provinces or cities in China from 2017 to 2020. All 4,414 non-duplicated *enterococci* strains were collected by the CHINET surveillance system, including 2,316 *Enterococcus faecium* strains and 2,098 *Enterococcus faecalis* isolates, stored in 40% glycerol broth and frozen at −80°C. The hospitals in the CHINET surveillance system collect bacteria every year, and the staff numbers in hospitals randomly collect bacteria in the process. The distribution of 4,414 isolates was 1,222 in 2017, 1,411 in 2018, 1,277 in 2019, and 504 in 2020. All strains isolated from children and adults accounted for 1.6 and 98.4%, respectively. Among them, the main sources of specimens were urine (64.4%), bile (12.2%), pleural and ascites (12.2%), blood (8.5%), wounds (6.6%), and respiratory tract specimens (5.3%). *E. faecalis* ATCC 29212 was used as a quality control strain.

### Antimicrobial Susceptibility Testing

The antimicrobial susceptibility to fosfomycin was determined using the agar dilution method by Mueller-Hinton agar (MHA) supplemented with 25 mg/L glucose-6-phosphate (G6P) and the results were interpreted by the Clinical and Laboratory Standards Institute (CLSI) M100 31th Edition guideline. To interpret the susceptibility of *E. faecium* to fosfomycin, values corresponded to the breakpoints of the urinary tract infection *E. faecalis* in CLSI (sensitivity ≤ 64 mg/L; resistant ≥ 256 mg/L) (Clinical and Laboratory Standards Institute, 2020).

### Detection of Fosfomycin Resistance Genes

The fosfomycin resistance genes were detected and confirmed by polymerase chain reaction (PCR) using the primers listed in Table 1. The PCR products were sequenced by the MAP BIOTECH company (Shanghai, China) and the sequences were aligned by BLAST^1^.

**TABLE 1:** PCR primers of genes.

Gene primer	Sequences (5′→3′)	Product size (bp)	Tm (°C)
*fos*B-F	CAGAGATATTTTAGGGGCTGACA	312	53
*fos*B-R	CTCAATCTATCTTCTAAACTTCCTG		
*fos*X-F	TTGGGGGTGGGAAAGTTGATA	243	64
*fos*X-R	AACTGCAATCCAAAGGTCGT		
*mur*A-F	TCGCTTTATGCCGAAATCTT	1302	52
*mur*A-R	CGGCCACAAAAAGAAGGATA		
*fos*X-F-BamHI	CCC*GGATCC*ATGGAAGGAGAG GAAATGGAA		
*fos*X-R-XbaI	CGGCCCCC*TCTAGA*TTAATCTGTT GGTTCTTTTTGGTAG	243	64
**qPCR-Primer**			
*fos*X-F-RT	CTGTTGGTTCTTTTTGGTAGCGA		
*fos*X-R-RT	AAAGCTGGAGAAAAAGGGTT	130	60
*purk*-F-RT	TGGTGGCAGGAAATGGTCAA		
*purk*-R-RT	AGGCTCACTGCTTCTGCAAT	163	60

*Tm, annealing temperature.*

### Cloning of *fos*X Gene

To further elucidate the function of *fos*X, we selected the *fos*X fragment from a fosfomycin-resistant *E. faecium* (MIC = 512 mg/L) for cloning and cloned it into the shuttle vector plasmid pIB166. The recombinant plasmid pIB166_*fos*X was transformed into *Escherichia coli* DH5α competent cells, selected with chloramphenicol (5 mg/L). Later, the resulting plasmids were electroporated from *E. coli* DH5α into *E. faecium* BM4105RF competent cells, selected with rifampin (50 mg/L) and chloramphenicol (5 mg/L). The presence of *fos*X in *E. coli* and *E. faecium* transformants were confirmed by PCR and sequenced by the MAP BIOTECH company (Shanghai, China).

### Pulsed-Field Gel Electrophoresis

A pulsed-field gel electrophoresis analysis was performed using a CHEF mapper system (Bio-Rad, United States) (Murray et al., 1990). Agarose gel blocks were lysed with proteinase K at 20 mg/ml and digested with SmaI. The digested DNA was subjected to electrophoresis at 6 V/cm, 14°C, in a 1.0% agarose gel with pulse times of 1 to 20 s for 20 h. The banding patterns were interpreted using the criteria devised by Tenover et al. (Chen et al., 2014).

### Multilocus Sequence Typing

Genomic DNAs of *enterococci* strains were subjected to whole-genome sequencing using Illumina (Illumina, San Diego, CA, United States) short-read sequencing (150 bp paired-end reads). To ensure the quality of information analysis, the raw data is filtered using FASTX-Toolkit software^2^, assembled by the velvet V1.2.03 software^3^, and used glimmer software^4^ to predict genes. The functional annotation of genes was confirmed by searching the NCBI’s nr library^5^. Isolates were screened using the following seven housekeeping genes: adenylate kinase (*adk*), ATP synthase-alpha subunit (*atpA*), D-alanine ligase (*ddl*), glyceraldehyde-3-phosphate dehydrogenase (*gyd*), glucose-6-phosphate dehydrogenase (*gdh*), phosphoribosylaminoimidazole carboxylase ATPase subunit (*pur*K), and phosphate ATP-binding cassette transporter (*pst*S). The alleles and sequence types (STs) were determined *via* the MLST database^6^. The phylogenetic tree was performed by MEGA_X and iTOL^7^.

### Total RNA Extraction and Quantitative Real-Time PCR

A single colony was selected from the blood agar plate and added to brain heart infusion broth and incubated for 18 h at 37°C with shaking at 180 rpm. The bacterial culture was centrifuged at 12,000 rpm for 5 min to collect the supernatant. Total RNA was extracted using a Life Real kit (Life Real, Hangzhou, China) according to the manufacturer’s instructions. Thereafter, the RNAs were reverse transcribed to cDNA for qRT-PCR analysis in the light of the manufacturer’s manual of a commercial cDNA synthesis kit with a gDNA eraser (Takara, Dalian, China). The qPCR was performed on a Life Tech-ViiA7 qPCR multiplex reactions System (Life Technologies, United States) with specific primers (Table 1) for the PrimeScriptTM RT reagent Kit (Takara, Dalian, China). The relative expression levels of the *fos*X gene were normalized to the *pur*K reference gene. The quantification of the target genes was analyzed using the comparative threshold cycle 2^–△△Ct^ method (Zhang et al., 2020). All experiments were repeated in triplicate independently. The MIC range of the strains carrying the *fos*X gene to fosfomycin was 512–4,096 mg/L. Among them, the MICs of fosfomycin of HS2166, HS570, HS2069 and HS1440 were 512 mg/L, 1,024 mg/L, 2,048 mg/L, and 4,096 mg/L. The relative expression of the *fos*X gene of HS570, HS2069, and HS1440 isolates was normalized to that of the HS2166 strain.

### Next Genome Sequencing

To evaluate the difference in antibiotic resistance of *enterococci* carrying the *fos*X gene with different levels of fosfomycin resistance, we extracted DNA from the HS2166, HS570, HS2069, and HS1440 strains and performed next genome sequencing according to the precious method (Xu et al., 2013). Raw sequences were filtered using FASTX-Toolkit (see text footnote 2) and assembled into contigs using the velvet V1.2.03 program. The Comprehensive Antibiotic Database^8^ was used to identify antibiotic resistance genes. The replicon sequences were identified by PlasmidFinder 2.1^9^. The Virulence Factors Database^10^ was used to identify virulence genes.

### Ethics Statement

The study protocol was approved by the Institutional Review Board of Huashan Hospital, Fudan University (no. 2020-040).

## Results

### Antimicrobial Susceptibility Testing and Detection of Fosfomycin Resistance Genes

Among the 4,414 isolates, the rate of fosfomycin-resistant *enterococci* was 3.5% (153/4414). Fosfomycin was very active against *enterococci* (MIC_50_ and MIC_90_ were both 50 mg/L; the median MIC value was 50 mg/L). Of 153 fosfomycin-resistant clinical isolates, *E. faecium* and *E. faecalis* accounted for 77.1% (117/153) and 23.5% (36/153), respectively. The MIC range of fosfomycin against 153 fosfomycin-resistant strains was 512–4,096 mg/L, of which MIC_50_ and MIC_90_ were 2,048 and 4,096 mg/L, and the median MIC value was 2,048 mg/L. The PCR results showed that the strains carrying the *fos*B and *fos*X genes were 33.8% (52/153) and 65.6% (101/153) in the current study, respectively.

### Cloning of *fos*X Gene, Pulsed-Field Gel Electrophoresis, and Multilocus Sequence Typing

**FIGURE 1:**
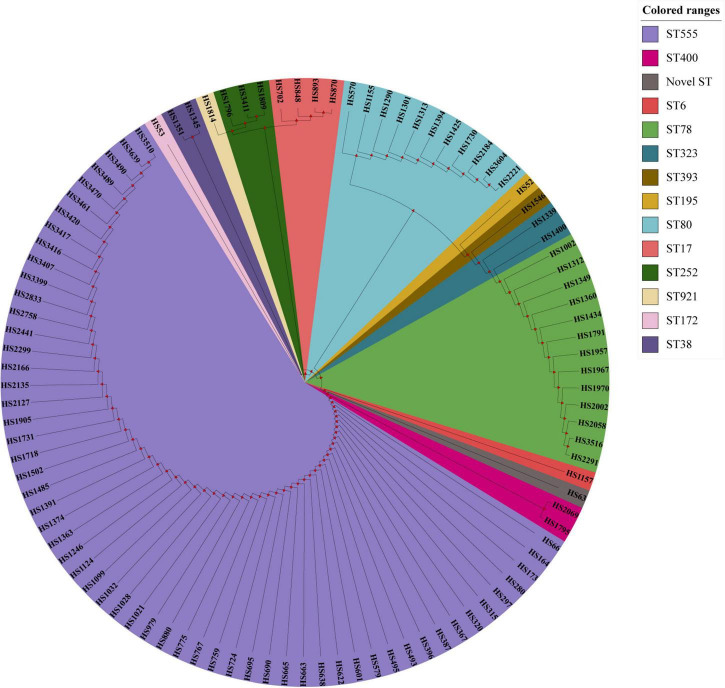
The MLST results of 101 *E. faecium* carrying *fos*X gene.

The *fos*X gene was cloned into *E. faecium* BM4105RF by cloning experiment, and the results revealed that the fosfomycin MICs of *E. faecium* BM4105RF transformant carrying *fos*X was 128 mg/L while the recipient and donor strains had fosfomycin MICs of 32 and 2,048 mg/L, indicating that *fos*X could be the expression in *E. faecium*. According to the results of PFGE, the *fos*X carrying *E. faecium* isolates were grouped into 48 pulse types. The top five pulse types were named ENT-27, ENT-15, ENT-29, ENT-8 and ENT-30, and the percentage were 8.9% (9/101), 5.9% (6/101), 4.9% (5/101), 4% (4/101), and 4% (4/101). The 101 *fos*X carrying *E. faecium* isolates in this study were categorized into fourteen different STs (ST555, ST6, ST17, ST38, ST78, ST 80, ST 172, ST195, ST252, ST323, ST393, ST400, ST921, and one novel ST). The top five STs were ST555, ST78, ST80, ST17, and ST252, and the percentage were 57.4% (58/101), 12.8% (13/101), 10.9% (11/101), 4% (4/101), and 3% (3/101). The minimum spanning tree analysis showed that seven groups were formed by *enterococci* STs by MEGA_X and iTOL analysis. The MIC of ST555, ST78, ST17, and ST252 *E. faecium* to Fosfomycin could reach 4,096 mg/L. 94% (95/101) of *E. faecium* belonged to the CC17 clonal complex, and the MIC of *E. faecium* to Fosfomycin can reach 4,096 mg/L. The fosfomycin MIC of 6% (6/101) of the *E. faecium* belonging to the non-CC17 clonal complex could reach 2,048 mg/L. In terms of geographical distribution, the distribution of 58 isolates of ST555 *E. faecium* was 44 in Shanghai, five strains in Beijing, two strains in Anhui, two strains in Hubei, two strains in Tianjin, one strain in Henan, one strain in Gansu, and one strain in Jiangsu province. Four ST17 *E. faecium* isolates were only distributed in Shanghai, and other types were scattered. The results of PFGE, MLST, and phylogenetic tree were summarized in supplement material and Figure 1.

### Quantitative Real-Time PCR

**FIGURE 2:**
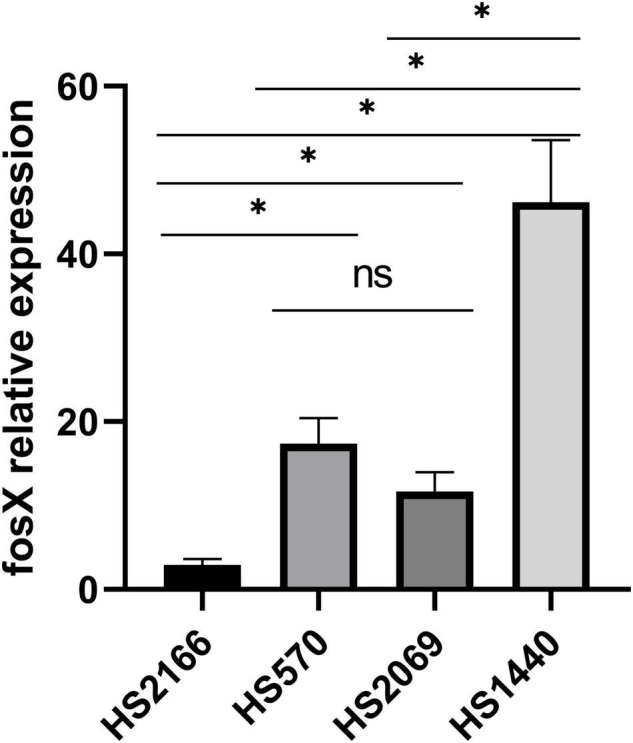
The relative expression of *fos*X gene in HS2166, HS570, HS2069, and HS1440.

Due to the presence of *fos*X in *E. faecium* with different fosfomycin MICs, we determined the relationship of *fos*X expression levels of different fosfomycin MICs of *fos*X carrying fosfomycin-resistant *E. faecium*. The fosfomycin MIC of four *E. faecium*, named HS2166, HS570, HS2069, and HS1440 were 512, 1,024, 2,048, and 4,096 mg/L. Interestingly, *fos*X expression of fosfomycin-resistant strain (HS2166, MIC = 512 mg/L) was lower than that of HS570, HS2069, and HS1440 isolates which showed fosfomycin MICs 1,024, 2,048, and 4,096 mg/L, and the *fos*X relative expression of HS570 and HS2069 strains showed no significant difference (Figure 2). Overall, the qPCR results showed that with the enhancement of the resistance to fosfomycin of *E. faecium*, the relative expression level of the *fos*X gene increased correspondingly.

### Next Genome Sequencing

**FIGURE 3:**
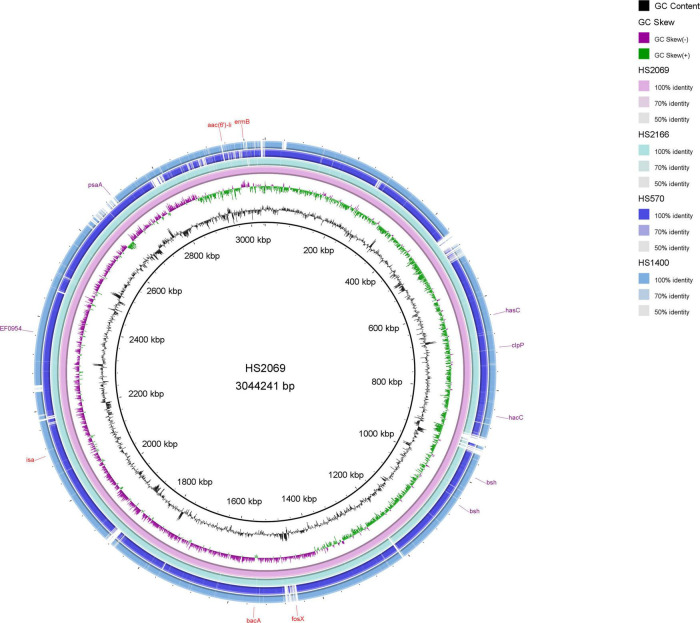
The circle map of HS2069, HS2166, HS570, and HS1440.

The four next genome sequencing results of HS2166, HS570, HS2069, and HS1440 strains were listed in Figure 3 and Table 2. The antibiotic resistance genes of the HS2166 strain with fosfomycin MIC of 512 mg/L were *aac(6’)-Ii*, *erm*B, *dfr*G, *dfr*F, *inu*A, *efm*A, and *fos*X genes. There were *aac(6’)-Ii*, *aph(3’)-IIIa*, *efm*A, *erm*B, *tet*(45), *tet*U, and *fos*X genes in the HS570 strain with fosfomycin MIC of 1,024 mg/L. The drug resistance genes in the HS2069 strain with fosfomycin MIC of 2,048 mg/L were *aac(6’)-Ii*, *bac*A, *isa*, *erm*B, and *fos*X genes. The resistance genes in HS1440 strain with fosfomycin resistance level of 4,096 mg/L were *aac(6’)-Ii*, *ant*(6)-Ia, *efm*A, *dfr*G, *erm*B, *erm*T, *tet*(45), *inu*A, and *fos*X genes. The repUS15 and rep2 plasmids were present in all four strains, and the repIIa plasmid was present in HS2166, HS570, and HS2069. The plasmids in HS1400 isolate with fosfomycin MIC of 4,096 mg/L were repUS12, repUS15, repUS43, rep2, and rep29, and the virulence genes in the HS1400 strain were *has*B, *has*C, *cap*8D, and *kfi*D. Interestingly, no virulence gene was detected in the HS2166 strain with fosfomycin MIC of 512 mg/L. Only one virulence gene named *bop*D was detected in HS570 isolate with fosfomycin MIC of 1,024 mg/L.

**TABLE 2:** The comparative genome sequences in HS2166, HS570, HS2069, and HS1440 strains.

Strain	HS2166 (Fosfomycin MIC 512 mg/L)	HS570 (Fosfomycin MIC 1024 mg/L)	HS2069 (Fosfomycin MIC 2048 mg/L)	HS1400 (Fosfomycin MIC 4096 mg/L)
Size (bp)	2982542	3043392	2905269	2982542
G+C (%)	37.7	37.5	37.8	37.7
No. of contigs	162	183	137	150
Median sequence size	8385	6551	7071	7065
N50 value	41952	42274	54021	47858
L50 value	20	23	16	16
Resistance genes	*aac(6’)-Ii; erm*B*; dfr*G,*dfr*F*; lnu*A*; fos*X*; efm*A	*aac(6’)-Ii,aph(3’)-IIIa; efm*A*; erm*B*; tet*(45),*tet(U)*	*aac(6’)-Ii; bac*A*; fos*X*; erm*B*;Isa*	*aac(6’)-Ii,ant*(6)*-Ia; efm*A*; dfr*G*; fos*X*; tet*(45)*; lnu*A*; erm*B, *erm*T
Replicon	repIIa; repUS15; rep2	repUS12; repUS15; rep14a; rep2; rep17; repIIa	repUS15; rep2; repIIa	repUS12; repUS15; repUS43; rep2; rep29
Virulence gene(s)	none	*bop*D	*has*C*; clp*P*; EF0954; bsh; psa*A	*has*B*; has*C*; cap8*D*; kfi*D*;*

## Discussion

Due to the unique mechanisms of action, fosfomycin exhibits significant antimicrobial activity against a broad spectrum of pathogens, including *enterococci* (Mendoza et al., 1980; Bernat et al., 1997). The fosfomycin resistance rate of *enterococci* isolated from South Africa was 1% (72/725) (Sahni et al., 2013). Zhang et al. (2020) reported that the fosfomycin resistance rate of *enterococci* isolated from China was 2.6% (20/761). The fosfomycin resistance rate of *enterococci* isolated from the United States was 1.3% (12/890) (Guo et al., 2017). In this study, 3.5% (153/4,414) of the *enterococci* were resistant to fosfomycin.

Little was known about the resistance mechanism of *enterococci* to fosfomycin in epidemiological research, and the most of genes that have been reported to be plasmid-mediated resistance to fosfomycin were *fosB*, with a G+C content of 27%, and were mainly distributed in *staphylococcus*, *enterococci*, and *bacillus* (Chen et al., 2014; Fu et al., 2016; Guo et al., 2017; Shinde et al., 2017; Ziwei et al., 2019; Xu et al., 2020). The *fos*B gene developed resistance to fosfomycin by encoding Mg^2+^-dependent sulfhydryl transferase, while the *fos*X was by encoding Mn^2+^-dependent epoxidase (Roberts et al., 2013). Recently, there are reports that the *fos*X gene was detected in *Acinetobacter baumannii, Klebsiella pneumoniae*, and *E. faecium* (Zhang et al., 2020; Kashefieh et al., 2021; Leite et al., 2021), indicating that the *fos*X gene is not unique to Gram-positive bacteria. The MICs of *fos*X-positive fosfomycin-resistant *A. baumannii* and *K. pneumoniae* were 128 and ≥ 200 mg/L, respectively (Kashefieh et al., 2021; Leite et al., 2021). Meanwhile, the MIC of fosfomycin-resistant *E. faecium* carrying the *fos*X gene was ≥ 512 mg/L (Zhang et al., 2020). In the current study, the MIC range of *fos*X-positive *E. faecium* was 512–4,096 mg/L.

Notably, in the report of Zhang et al., *fos*X, *fos*B genes, and *mur*A mutation can coexist in fosfomycin-resistant *enterococci* (Zhang et al., 2020). Mutation of MurA protein exists in *A. ba* carrying *fos*X gene with fosfomycin MIC of 128 mg/L (Leite et al., 2021). Unlike previous studies, the *fos*X gene was exclusively present on fosfomycin-resistant *E. faecium*. More importantly, this study was the first time to clone the *fos*X gene in *enterococci* and proved that the *fos*X gene can be expressed in *E. faecium*, which mediates the reduction of the bacteria’s sensitivity to fosfomycin, indicating that these genes might be the primary factors mediating the resistance of *E. faecium* against fosfomycin.

As previously reported, the PFGE band patterns of 20 *fos*X-positive fosfomycin-resistant *enterococci* isolates showed 16 closely related isolates that exhibited ≥ 80% similarity (Zhang et al., 2020). In the current study, the PFGE patterns of 101 *enterococci* carrying the *fos*X gene showed 48 related strains that exhibited ≥ 80% similarity. A small number of isolates showed a high degree of homology, indicating that the possibility of genetic correlation between them was extremely small. The most frequent sequence type of enterococci carrying the *fos*X gene was the ST78 type in the previous studies (Zhang et al., 2020). Differently, ST555 (59/101) was the main clonal type in this study. The CC17 clonal complex *E. faecium*, which usually carries a virulence island and causes rapid dissemination in hospitals (Mancuso et al., 2021), belongs to a clonal complex closely related to nosocomial infection (Fiore et al., 2019). In this research, 94% (95/101) strains of *E. faecium* carrying the *fos*X gene belonged to the CC17 clonal complex, 57.4% (58/101) of the *E. faecium* belonged to the ST555 type, and most of them were isolated from Shanghai in China. Therefore, monitoring the ST555 type *E. faecium* is of great significance to curb the spread of antibiotic-resistant *enterococci*.

It was reported (Zhang et al., 2020) that the susceptibility to fosfomycin of *enterococci* carrying the *fos*X gene included resistance and sensitivity. However, the differences were that the *enterococci* carrying the *fos*X gene detected in this article were all fosfomycin-resistant strains. In addition, among the fosfomycin-resistant strains, the MIC of the isolates to fosfomycin is related to the expression level of the *fos*X gene, indicating that the function of the FosX resistance protein needs further verification.

In *enterococci*, the mobile genetic elements could account for 25% of the entire bacterial genome with strong plasticity (Hollenbeck and Rice, 2012). The fosfomycin resistance proteins FosX and FosA in pathogenic microorganisms are related to a catalytically promiscuous progenitor encoded in a *phn* operon in *Mesorhizobium loti* (Fillgrove et al., 2007). Chen et al. (2019) found that the *fos*A3 gene in clinical KPC-producing *Klebsiella pneumoniae* isolates collected from Zhejiang in China had an identical genetic background, *IS*26-*tet*R-*cad*C-*orf*1-*fos*A3-*IS*26, which is the same as that of the *fos*A3-positive plasmid pFOS18 in China. No other drug resistance genes have been reported in *E. faecium* carrying the *fos*X gene before. Parra-Flores et al. (2021) reported that ampicillin-resistant *Listeria monocytogenes* strains carried the *fos*X, *tet*A, and *tet*C resistance genes. In this study, *fos*X, *aac(6’)-Ii*, and *erm*B resistance genes co-existed in the genomes of *E. faecium* strains. Through the expression of the *fos*X gene was previously reported to be associated with the virulence factors hpy and prfA (Scortti et al., 2018), no such virulence genes were found in this study. The *bop*D (biofilm formation) virulence gene belongs to the type III secreted protein and exists in the HS570 strain with fosfomycin MIC of 1,024 mg/L. A large number of gram-negative pathogenic bacteria secrete virulence factors *via* the type III secretion system during infection of host cells (Nogawa et al., 2004). The capsular genotype (*has*ABC) (Kaczorek et al., 2017), K5-specific UDP-glucose dehydrogenase (*kfi*D) (Muñoz et al., 1998), caseinolytic peptidase P (*clp*P) (Jing et al., 2022), bile resistance(*bsh*) (Parra-Flores et al., 2021), pneumococcal surface adhesion A (*psa*A) (Hu et al., 2021), and polysaccharide biosynthesis (*cap*8D) (Ali et al., 2016) were detected from *E. faecium* in this study. Unfortunately, the effect of virulence factors on fosfomycin-resistant bacteria is unclear and needs to be explored by researchers.

It was worth noting that the previous report of the *fos*X gene in *enterococci* had only appeared in Zhejiang, China, which reported that 20 *fos*X-positive fosfomycin-resistant *enterococci* were screened out of 790 *enterococci* isolates. This study was the first time to screen large-scale fosfomycin resistance genes in *enterococci* isolated from 62 hospitals in 26 provinces or cities in China and found 101 fosfomycin-resistant strains carrying the *fos*X gene among 4,414 *enterococci* clinical strains.

## Conclusion

In summary, 153 fosfomycin-resistant strains were screened out of 4,414 *enterococci* clinical isolates, and mechanisms were explored. Our finding described that *fos*B and *fos*X genes were the major resistant mechanism of *enterococci* to fosfomycin. In addition, the *fos*X gene could increase the resistance of bacteria to fosfomycin in *E. faecium* bacteria. FosX overexpression was associated with high-level fosfomycin resistance in *E. faecium* clinical isolates. Moreover, the 101 *fos*X carrying *enterococci* showed no obvious clonal transmission by PFGE analysis, and the dominant sequence type in them was ST555. There were 14 groups of 101 *E. faecium* carrying *fos*X were formed by *E. faecium* STs by MEGA-X and iTOL analysis. Therefore, it is necessary to continuously monitor fosfomycin resistance and its mechanisms.

## Data Availability Statement

The datasets presented in this study can be found in online repositories. The names of the repository/repositories and accession number(s) can be found in the article/supplementary material.

## Author Contributions

FH and YG designed the study. LX, QS, and RH collected clinical samples. LX, XX, and JW performed the experiments. LX, YG, and FH analyzed the data and wrote the manuscript. All authors contributed to the article and approved the submitted version.

## Conflict of Interest

The authors declare that the research was conducted in the absence of any commercial or financial relationships that could be construed as a potential conflict of interest.

## Publisher’s Note

All claims expressed in this article are solely those of the authors and do not necessarily represent those of their affiliated organizations, or those of the publisher, the editors and the reviewers. Any product that may be evaluated in this article, or claim that may be made by its manufacturer, is not guaranteed or endorsed by the publisher.

## Footnotes

1
http://blast.ncbi.nlm.nih.gov/Blast.cgi

2
http://hannonlab.cshl.edu/fastx_toolkit

3
https://www.ebi.ac.uk/~zerbino/velvet

4
http://ccb.jhu.edu/software/glimmer/

5
ftp://ftp.ncbi.nih.gov/blast/db

6
http://efaecium.mlst.net/

7
https://itol.embl.de/

8
https://card.mcmaster.ca/analyze

9
https://cge.food.dtu.dk/services/PlasmidFinder

10
http://www.mgc.ac.cn/VFs
